# Prevention of Asbestos Exposure in Latin America within a Global Public Health Perspective

**DOI:** 10.5334/aogh.2341

**Published:** 2019-03-29

**Authors:** Eduardo Algranti, Juan Pablo Ramos-Bonilla, Benedetto Terracini, Vilma S. Santana, Pietro Comba, Roberto Pasetto, Agata Mazzeo, Fulvio Cavariani, Andrés Trotta, Daniela Marsili

**Affiliations:** 1 Serviço de Medicina, FUNDACENTRO, São Paulo, BR; 2 Department of Civil and Environmental Engineering, Universidad de los Andes, Bogotá, CO; 3 University of Turin (Now Retired), Turin, IT; 4 Instituto de Saúde Coletiva, Universidade Federal da Bahia, Salvador, BR; 5 Department of Environment and Health, Istituto Superiore di Sanità, Rome, IT; 6 School of Arts, Humanities, and Cultural Heritage, University of Bologna, Bologna, IT; 7 Centro Regionale Amianto Lazio Dipartimento di Prevenzione, Unità Sanitaria Locale, Viterbo, IT; 8 Instituto de Salud Colectiva (ISCo)/Institute of Collective Health, Universidad Nacional de Lanús (UNLa)/National University of Lanús, Buenos Aires, AR

## Abstract

**Background:**

Asbestos consumption in Latin America (LA) amounts to 10% of yearly global production. Little is known about the impact of asbestos exposure in the region.

**Objective:**

To discuss scientific and socio-economic issues and conflicts of interest and to summarize epidemiological data of asbestos health effects in LA.

**Discussion:**

Recent data on chrysotile strengthened the evidence of its carcinogenicity and showed an excessive risk of lung cancer at cumulative exposure levels as low as 1.5 fibre-years/ml. Technology for substitution is available for all asbestos-containing products and ceasing asbestos production and manufacturing will not result in unemployment and loss of income, except for the mining industry. The flawed arguments used by the industry to maintain its market, both to the public and in courtrooms, strongly relies on the lack of local evidence of the ill effects and on the invisibility of asbestos-related diseases in LA, due to the limited number of studies and the exposed workers’ difficulty accessing health services. The few epidemiological studies available show clear evidence of clusters of mesothelioma in municipalities with a history of asbestos consumption and a forecasted rise in its incidence in Argentina and Brazil for the next decade. In Brazil, non-governmental organizations of asbestos workers were pivotal to counterbalance misinformation and inequities, ending recently in a Supreme Court decision backing an asbestos ban. In parallel, continuous efforts should be made to stimulate the growth of competent and ethical researchers to convey adequate information to the scientific community and to the general public.

## Introduction

Occurrence, industrial production, and use of asbestos represent a critical issue for public health at a global level, both currently and in the long term. Two main WHO statements confirm that discontinuing asbestos use is the most efficient way to prevent asbestos-related diseases (ARDs) due to occupational and environmental exposure [1,2]. These statements subsume the ascertained carcinogenicity of all forms of asbestos to humans [3], including chrysotile, and failure in using the ratification of the ILO 162 Convention on asbestos as a justification or endorsement for stopping the use of asbestos [4]. Nevertheless, hurdles in translating scientific evidence into health prevention policy and multi-sectorial interventions to reduce the impact of ARDs have been documented by the international scientific literature, which has also considered the economic affairs of transnational and national asbestos industry and related conflicts of interest [5,6,7,8,9,10,11,12].

In the last decade, Latin America (LA), where most countries still use asbestos (only Argentina, Chile, Uruguay, Honduras and, very recently, Brazil have banned its use so far), contributed with approximately 10% to the world’s asbestos production and consumption (approximately 200 thousand tons per year out of approximately 2 million tons per year) [13,14,15]. In this frame, Brazil had a predominant role in continuing asbestos extraction, use and export in LA, and it well represents the societal impacts of asbestos, including on labor, industry, environmental and public health, urban planning, media, and courtrooms. The LA scientific community is thus required to disseminate scientific findings effectively in order to provide appropriate tools for promoting and strengthening prevention actions. This task can benefit from scientific cooperative networks including multidisciplinary know-how and expertise from countries that have reached different levels in policy intervention towards asbestos. In this perspective, the present paper aims to discuss critical issues on asbestos from the scientific, socio-economic, and epidemiological points of view, in order to support the adoption of prevention measures and policies in LA countries and worldwide.

## The carcinogenic risk of asbestos with a special focus on chrysotile

A detailed account of the acquisition of data on cancer risk associated to asbestos exposure has been discussed in a previous paper [7]. The most recent IARC evaluation was conducted in 2009 [3]. It concluded that there was sufficient evidence that all forms of asbestos, including chrysotile, crocidolite, amosite, tremolite, actinolite, and anthophyllite, are carcinogenic to humans. The evidence of a causal relationship was judged to be sufficient for mesothelioma and cancers of the lung, larynx, and ovary and limited for cancers of the pharynx, stomach, and colorectum. With the exception of mesothelioma, cancer in these target organs is multifactorial, so that in specific circumstances, assessing the role of asbestos may be problematic, particularly for lung cancer, given its frequency [16].

The burden of asbestos-related lung cancer can be estimated by different methods, yielding variable results [17,18,19]. The average ratio of lung cancers to mesotheliomas in chrysotile exposed cohorts was estimated in 6.1 [18]. The number of lung cancer cases attributable to asbestos calculated with the methods mentioned above is far higher than those provided by studies based on official registries [20]. Consequently, the total burden of asbestos-related cancers can be grossly underestimated.

In the recent IARC evaluation, the Working Group concluded that, for lung cancer, it was “not possible to draw any firm conclusions concerning the relative potency of chrysotile and amphibole fibres” [3]. In a subsequent meta-analysis of 19 studies which also considered the quality of exposure assessment, there appeared to be little difference in the slopes of the curves describing risk for cumulative exposure to chrysotile compared to amphibole fibres [21]. In contrast, for mesothelioma, the IARC Working Group judged that there was “considerable evidence” of different potency among asbestos fibre types, with chrysotile having lower potency than amphibole asbestos [3]. This does not detract from the evidence that chrysotile induces mesothelioma, as shown by a number of studies in many countries: the most recent probably being an update of the mortality among ex-workers in the chrysotile quarry of Balangero [22].

Reviews denying the carcinogenicity of chrysotile, such as Yarborough [23], have not considered all the scientific epidemiological and experimental evidence that is available. Another argument frequently mentioned as suggesting innocuity of chrysotile is that chrysotile is cleared from the lung parenchyma more rapidly than amphiboles, so that more amphiboles than chrysotile are detected in autopsy studies [24]. However, given the long latency period of cancer, inferring that chrysotile is innocuous because of its scarcity in autopsy material is a twisted conclusion. Indeed, a very recent study based on patients in which asbestos bodies in human biological material had been counted in two occasions, years apart, suggests that chrysotile clearance may not proceed as quickly as previously thought [25].

The excess risk of lung cancer in chrysotile-exposed workers has been demonstrated in mining [26] and in the manufacturing of textiles [27,28]. A number of studies including asbestos mining, rubber and asbestos-cement (A/C) industries, published after the last IARC evaluation, lend further support to longstanding conclusions [29,30,31], including increasing rates and hazard ratios with increasing cumulative exposures [28,31]. Most of the cited studies refer to highly exposed chrysotile workers. However, Hein has shown an excess risk for workers whose cumulative exposure was less than 1.5 f-years/ml (e.g., 0.1 f/ml for 15 years or 0.5 f/ml for 3 years) [27].

After the last IARC evaluation, a number of studies added knowledge to lung cancer risk at low cumulative asbestos exposures and to the effect of smoking. An analysis of the 19 studies mentioned above [21] estimated relative lung cancer risks between 1.01 and 1.03, and provided estimates of 1.13 and 1.30 for cumulative exposure levels of 4 and 40 f-y/ml, respectively [32]. A very recent pooled analysis of 14 case-control studies for a total of more than 17,000 cases and 21,000 controls estimated odds ratios for ever exposure to asbestos of 1.24 (95% CI 1.18–1.31) in men and 1.12 (0.95–1.31) in women [33]. In the same study, in men, a series of case-control comparisons consistently showed a statistically significant increased risk for cumulative exposures of 1.2 f-years/ml. In cohort studies, meta-analyses, and pooled case-control analyses, the joint effect of asbestos exposure and smoking has been shown to be somewhere between additive and multiplicative [33,34,35].

Furthermore, some studies published after the IARC monograph confirmed the increased risk of cancer of the esophagus, stomach, and colorectum associated with occupational asbestos exposure. All three cancers were in excess in a general-population cohort in the Netherlands, with significant trends for gastric and colorectum cancers in men with exposure duration [36] and among Chinese chrysotile miners, with significant exposure-response trend for gastric cancer [37]. Elevated risk of colorectal cancer but not esophageal cancer was also reported in a study of workers at a factory in France that used both chrysotile and amphibole asbestos [38]. Elevated standardized incidence rates for esophagus, liver, and colorectal cancers were estimated in a cohort of French former asbestos reprocessing plant workers with an exposure duration of above 25 years [39]. In another French cohort study of men formerly exposed to asbestos, the hazard ratio for colon cancer was elevated [40]. In contrast, a mortality study of a pool of more than 50,000 Italian asbestos workers did not confirm an excess of digestive cancers [41].

Recent data have served to consolidate the carcinogenicity of chrysotile and to demonstrate increased lung cancer risk at low cumulative asbestos exposures. Data from recent studies support the evidence that the large intestine is an additional target organ. Importantly, early indications of a substantial differential in the risk of lung cancer according to the type of fibre have not been confirmed.

Comparing chrysotile and the amphiboles in their ability to produce cancer per unit of exposure may pose scientific interest. The caveat in this comparison is its irrelevance to public health. Chrysotile may indeed be “less carcinogenic” than amphiboles; however, in addition to its carcinogenic “potency,” the number of cancer cases produced by any environmental agent depends on the extent of opportunities for human beings to be exposed to it. Any relatively “low” carcinogenicity of chrysotile is largely balanced by the fact that, nowadays, over 200 thousand metric tons of this material is used in LA countries per year. Most workers exposed to asbestos in these countries are not protected by any strategy intended to reduce or eliminate exposure. Finally, the ability of chrysotile to produce cancer in experimental animals, apparently with the same potency of amphiboles, has never been disproved and cannot be dismissed in terms of public health.

## Economics

Many industrializing countries have been slow to reduce, let alone ban, the use of asbestos. The multiple factors at play include the low price and easy accessibility of asbestos, demand from the construction sector in emerging economies, and sustained efforts by the global asbestos industry and local companies to minimize its health risks.

In 2013, LA countries consumed 220,000 tons of asbestos, that is, 10% of the worldwide production [14]. The greatest users are Brazil, Colombia, and Mexico. In 2013, consumption was approximately 4,000–5,000 tons in Bolivia, Cuba, and Ecuador. In the three countries, consumption has tripled since 1990. It was less than 1,000 tons in all other LA countries [14,42]. Throughout the decades, LA countries have used almost exclusively chrysotile. However, during 1980–2003, Mexico and Argentina imported, respectively, a total of over 30,000 and over 10,000 tons of crocidolite and/or amosite from South Africa. Imports by Colombia, Chile, Brazil, Ecuador, Peru, and Cuba were between 1,000 and 8,000 tons [43].

Only in Brazil, to date, more than 6 million tons of asbestos have been used. In all countries, large quantities of asbestos remain as a legacy from past construction practices in many thousands of schools, homes, and commercial buildings, as well as in various industrial applications. Even in countries where a ban has been imposed, very little is known on remediation trends as well as on practices intended to safeguard workers engaged in the removal of asbestos-containing products (ACPs) and the handling of the resulting waste material.

Mexico has never produced asbestos. However, since 1970, consequent to increased regulation of asbestos in Europe and in the United States, a massive transfer of asbestos-processing enterprises has taken place. By 2001, 1,881 Mexican companies were importing asbestos. In the 1990s, annual asbestos consumption was in the order of 38,000 tons, 50% from Canada and 12% from Brazil. In the current decade, yearly consumption has fluctuated between 7,000 and 17,000 tons, and imports from Canada have ended. Despite the drop in imported asbestos, exports of asbestos goods from Mexico tripled between 1992 and 2000 [43]. In 1992, the destination of almost all exports was the United States, whereas, ten years later, almost one third of the exports went to Central American countries. Employment data relating to the asbestos industry are very limited. Early in the new millennium, asbestos-using companies in the Valley of Mexico employed approximately 5,000 workers. An additional 15 A/C industries insured over 7,000 workers [44,45].

In Colombia, production at the Las Brisas mine was around 5,000 tons per year. Johns Manville Corporation abandoned the mine in 1998, and production ceased shortly thereafter [46]. In fact, most of the asbestos used in Colombia has been imported from Brazil and Canada. In the last couple of decades, total yearly consumption fluctuated around 20,000 tons [42]. Almost all asbestos goods manufactured in the country are for internal use, and exports have been very limited. The use of A/C has been extensive: according to the reference source, 1.5 to 5.0 million dwellings are covered by A/C and 40,000 km of pipes are made with A/C [47,48]. In 2009, of the enterprises associated with the Colombian System on Occupational Risks, 233 were involved in asbestos-related activities: 18 in vehicle construction/repair, 170 in demolition/excavation, and 33 in building, with 9,089 employees – 2,311 of them have been considered exposed to asbestos. The report does not inform completeness of the data collection and criteria for assessing workers’ exposure to asbestos [47].

Massive production of asbestos in Brazil started in 1967 [42]. Between 1970 and 1980, annual production jumped from 16 to 170 thousand tons, and consumption jumped from 37 to 195 thousand tons. In the new millennium, yearly production has reached 300 thousand tons, whereas yearly domestic consumption has fluctuated between 100 and 200 thousand tons. Exports began in the mid-1980s, went up to 100 thousand tons at the turn of the millennium, and have been in the order of 150 thousand tons in recent years [42]. Initially major destinations were LA countries, whereas in 2003, major export markets (in decreasing order) included Thailand, India, Iran, Indonesia, and Mexico. More than 99% of raw asbestos consumption goes to the A/C industry. In 2014, it was estimated that A/C production, used mainly in civil construction, generated around 800 million dollars per year, as A/C roof tiles are present in more than 25 million houses in Brazil. In 2015 Brazilian workers in the sector were estimated to be in the order of less than 4,000 [49].

Two documents on the possible impact of a ban in Brazil have produced contradictory results. The Construction Branch of the São Paulo Federation of Industries estimated that the sudden prohibition of the use of asbestos would produce a 69% cut in the offer of roof tiles, a 5–9% increase in the cost of popular dwellings, and the loss of jobs. According to the same document, the current technology based on the use of asbestos could not be adapted to alternative fibers [50]. Contrary conclusions were reached by a multidisciplinary study coordinated by the University of Campinas in which Brazil has made serious progress in replacing asbestos with alternative fibres [51]. The excess cost created by the use of alternative fibers would not exceed 10% (compensated by the ending of the costs caused by asbestos disposal), and the job loss would occur only in the sphere of asbestos extraction [51].

The asbestos-related economy in LA countries is a widespread and complex reality that determines, among other things, policy making. A growing number of scientific reports and position papers have increased awareness. In this frame, one more specific point should be mentioned, namely the attempts to provide figures on the economic cost to society caused by the environmental and health impacts of the use of asbestos. A recent publication has stated that banning asbestos in the various producer countries would neither affect employment nor their GDP. In addition, persistent use will result in high health, remediation, litigation, and compensation costs [52]. The publication “The Human and Finance Burden of Asbestos in the WHO European Region” [53] should also be mentioned. It reports the conclusions and recommendations of the WHO Member States Meeting aimed at supporting national efforts in developing and implementing national programs for the elimination of ARDs according to the ILO/WHO outline. The report describes methodologies and tools for estimating number of deaths, potential life-years lost, disability-adjusted life-years, and economic burden due to such diseases, underlying the importance of contributing to define and develop asbestos national profiles.

## Industry efforts to maintain the ongoing use of asbestos and to contradict causality of asbestos-related diseases

Currently, approximately 80% of the global population lives in countries where asbestos has not been banned. To justify the ongoing asbestos consumption, industry uses several arguments. These arguments include a potency difference between chrysotile and other asbestos types, the use of non-friable asbestos to prevent exposure, the absence of safe substitutes, and the promotion of a safe use of the material including a “responsible-use programme that is based on the controlled-use approach to regulating chrysotile.” These arguments can be found at the sites of the Chrysotile Institute and the International Chrysotile Association [54,55]. All these arguments were critically addressed in a previous publication [7]. We will discuss here additional arguments and strategies that have been used to delay regulatory interventions or lessen the potential health risks derived from asbestos use.

1. It has been argued that because the incidence of ARDs in a country or specific region is low, regulatory interventions should be delayed until a better understanding of the problem at the local level is reached [48]. As discussed in detail in other sections of this paper, a problem of under-diagnosis of ARDs has been documented in several countries of the world, which is frequently the consequence of having deficiencies in health surveillance or in the health system available (e.g., lack of properly trained medical professionals and difficult access to high-quality health services). Furthermore, one has to remember that the “absence of evidence is not evidence of absence,” something that is even more relevant for public health [56,57]. Thus, surveillance and diagnostic problems of ARDs should not be used as an excuse to delay regulatory interventions.
2. The industry argues that there are no local studies to prove that asbestos is producing adverse health effects or is exposing the population, or when the studies exist, it argues that local studies do not represent a holistic picture of the asbestos problem in the country and there is need of more studies [48,58]. Based on the large body of scientific evidence that exists on the adverse health effects resulting from asbestos exposure, requesting more studies at the local level as a required step that precedes regulatory interventions is unnecessary; on the contrary, it has a dilatory effect on these regulations. Furthermore, requesting more studies could shift the burden of proof to the local health and environmental authorities. Thus, instead of the asbestos industry having to prove that asbestos is “safe” or is being used under the “safe use” concept, governmental authorities have to prove that people are being exposed and that they are developing ARDs. This lack of evidence to support the “safe use” argument was recently documented in a literature review [59]. To counter this, it must be reiterated that it has been clearly proven that all forms of asbestos are carcinogenic to humans, that asbestos is a health risk worldwide, and that no additional evidence is required to support the implementation of regulations at the country level.
3. The industry (and in some cases congress members) argues that many workers and families depend on the asbestos industry for their income, as was shown recently in Colombia in a failed attempt to ban asbestos in this country [48]. This argument completely ignores the externalities caused by asbestos because of the economic and social/human costs resulting from ARDs for the entire society [52]. Furthermore, the argument ignores the premise that the common good is above private interests. A recent macroeconomic study conducted in Brazil has pointed that the impact of an asbestos ban would cut jobs only in the asbestos mining industry and create a transient rise in construction costs for a limited period of time [51].
4. The industry argues that asbestos is an occupational problem, not a public health problem [48]. This argument seems to lessen the extent and importance of the asbestos problem at the local and global levels, and denies that asbestos-containing products pose a risk of exposure and disease for any person, regardless of whether the person is a worker in an occupational setting, a person living in the same household of an asbestos worker, or a member of the general public. Currently, 2 million tons of asbestos are distributed around the globe each year [9,60], and most of this asbestos leaves the occupational setting in products with extended use, such as construction and friction products. For example, data obtained from documents of the asbestos industry in Colombia shows that in Colombia, more than 300 million square meters of asbestos-containing roof tiles were distributed over a period of approximately 65 years [48]. Furthermore, 375,000 heavy-duty vehicles, 165,000 public service passenger vehicles, and 3,190,000 passenger vehicles use asbestos-containing brake products [54]. Non-friable asbestos roof tiles experience a degradation of the encapsulation matrix in the long term, releasing the asbestos fibres [61], which may result in asbestos exposures beyond the occupational setting. Furthermore, manipulation of non-friable asbestos-containing products may also release asbestos in the short term, resulting in high personal exposures, as it has been shown for auto mechanics [62,63]. Considering the volume of asbestos distributed over the last several decades on a global scale, it is clear that a public health problem of great dimension has been created in LA.
5. The asbestos industry has been using specific arguments in the courts in order to dismiss causal links between occupational and environmental exposures and occurrence of mesothelioma and other ARDs. We highlighted some flaws in these arguments in our previous paper [7]. A recent paper by Terracini et al. [64], specifically confutes the notion according to which recent exposures to asbestos do not contribute to causation of mesothelioma. The judicial implication of this notion would be that only managers in charge of factories where the patient was exposed in the early years of his career might be regarded as liable; however, most of them would be deceased at the time of recent trials, and thus not prosecuted. Furthermore, other strategies are used challenging the diagnosis of asbestosis and other ARDs on the grounds of “questionable asbestos exposure” or that the industry “provided respirators to workers” or that “differential diagnosis was not addressed” or that “the disease was a consequence of other risk factors,” such as smoking. Often, industry experts try to induce the court to demand objective evidences of exposure, such as fibre counts in bronchial lavage fluids or in lung tissue biopsies. Diagnoses of asbestosis and asbestos-related cancers are made by integrating a relevant occupational history, a latency period, and an image method with suggestive findings. The existence of other previously diagnosed cases in the industry is also a strong support for the diagnosis. These criteria are used worldwide [65,66]. Invasive procedures are needed only in instances where the occupational history is not clear or there are clinical signs that suggest the search for a differential diagnosis. It should be stressed that attribution based on fiber counts in lung tissue may be misleading, given the long latency time of ARDs and fiber clearance dynamics [67].

## Epidemiological studies on mesothelioma in Latin America

Until now, regular reporting of both exposure and ARDs remains poorly implemented in LA, limiting precision of morbidity and mortality estimates.

The main reason for using mesothelioma as an indicator of asbestos exposure in a society relies on the high specificity of the association with asbestos [68]. In a recent bibliometric study on asbestos and mesothelioma, 6,907 publications were found corresponding to 65 countries, of which 28 had more than 12 articles. Brazil was the only LA country listed, with at least 22 articles [69].

Reviewing asbestos and mesothelioma publications from LA countries, we found several descriptive case series studies [70,71,72,73,74,75,76,77,78]. Few studies presented mesothelioma mortality estimates for the region, presumably because data to all LA countries were unavailable, unreported, or limited in quality and coverage. Nishikawa et al. [79], using the WHO database from 1996–2005, calculated age-adjusted average annual mesothelioma mortality to be 3.1/1,000,000 in Chile, 2.5 in Argentina, 2.3 in Uruguay, 2.2 in Mexico, 0.6 in Cuba, and only 0.5 in Brazil and in Ecuador.

In recent years, national estimates of mesothelioma mortality in LA have been described, limited to a few countries. In Brazil, according to the Brazilian Mortality Information System (SIM), overall mesothelioma mortality was 0.56/1,000,000 in 1980 and increased to 1.01/1,000,000 in 2003, corresponding to an 80.4% growth in 23 years for the entire population [80]. Recent estimates [81] have shown a persistent increase of mesothelioma mortality over time for men, reaching an average of 1.0/1,000,000 between 2005 and 2010, close to the estimate for women (0.96/1,000,000), revealing a very small male to female ratio. Algranti et al. [82], analyzed mesothelioma deaths from 2000 to 2012, for adults 30 years of age or older. The authors reported that in spite of a significant increasing trend in age-adjusted standardized mortality found in the state of São Paulo, where almost half of the asbestos industries were settled and specialized healthcare services were available, this was not replicated for the entire country. Clusters of mesothelioma cases were found in Brazilian municipalities that had formerly harbored A/C industries [82].

After decades of heavy asbestos use, Argentina instituted a national ban in 2001 [72]. Using WHO data, Park et al. [83], reported that from 1994 to 2008, a total of 1,065 mesothelioma deaths were registered in Argentina – 97 per year in average. Using data from the Argentina Mortality Information System from 1980 to 2013 and limiting the data to individuals aged 15 years or older, the age-adjusted mesothelioma mortality was 3.04/1,000,000 in 1980, increasing to 5.62/1,000,000 in 2013, which corresponded to a 84.1% growth, with a clear linear trend after 1997 [84].

Based on the historical asbestos consumption or in an age-period-cohort effect, forecasting of mesothelioma mortality is available for Brazil [82] and Argentina [85]. Findings from both studies show an increasing trend in mortality until 2026 in Brazil and 2020 in Argentina.

A prospective study of pleural mesothelioma in Mexico involving 119 cases and 353 controls disclosed increasing odds ratios by increasing levels of exposure [45]. Attributable risks to asbestos exposure were estimated at 83.2% for the exposed population and 44% for the general population.

Some studies have addressed how mesothelioma cases are captured across multiple data sources to estimate underreporting. In Brazil, mesothelioma cases (n = 217) recorded in death certificates from the city of Rio de Janeiro between 1979 and 1994 were tracked down to find the corresponding diagnosis from histopathology and medical records, enabling the estimation of agreement level of diagnosis across all data sources [86]. Only a few cases (n = 31) could be paired with data from other databases. Among them, agreement between mesothelioma diagnosis from death certificates and histopathology was 35.5%, and with medical records, 59.3%. In Mexico, Aguilar-Madrid et al. [45], also found low agreement (29.0%) when comparing mesothelioma diagnosis from immunohistopathology with codes recorded in death certificates. These is strong evidence that underreporting of mesothelioma may be generalized in LA countries.

Pasetto et al. [19], using an approach based on the Population Attributable Fraction [87], estimated the burden of mesothelioma, lung, larynx, and ovary cancers attributable to occupational asbestos exposures in Argentina, Brazil, Colombia, and Mexico. Although the method is based on several assumptions that could not be checked and also on the reliability of national vital statistics, a sizeable number of asbestos-related cancers was estimated. No estimates of mesothelioma mortality were found for other LA countries.

**Figure 1:**
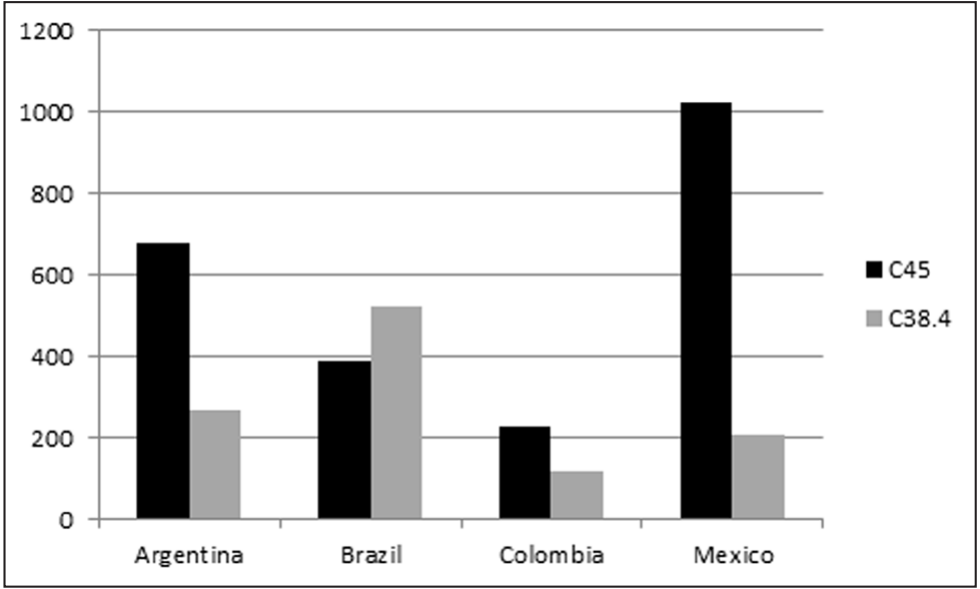
Cumulative number of deaths from Mesothelioma (C45) and Cancer of the Pleura (C38.4) in the period 2005–2009 in Argentina, Brazil, Colombia and Mexico [data from reference [19].

Until the beginning of this century, asbestos consumption in absolute volume was 20 times higher in Brazil compared to Argentina, and 5 to 10 times higher than in Mexico [42]. Based on the WHO mortality database used by Pasetto et al. [19], Figure 1 shows the number of mesothelioma (ICD 10 C45) and cancer of the pleura cases (ICD 10 C38.4) as the underlying causes of death registered from 2005–2009 in Argentina, Brazil, Colombia, and Mexico. The number of mesotheliomas was much higher in Argentina and Mexico, in spite of a heavier Brazilian consumption. It also contrasts with the age-adjusted mortality rates from mesothelioma, 3 to 5 times higher in Argentina when compared to those from Brazil [82,84].

The explanation that these mesothelioma mortality differences results from the fact that some LA countries have better worker protection against asbestos occupational exposure and chrysotile use is safe is implausible [88]. This could be the evidence of large underreporting caused by low coverage, lack or poor training for case assessment, and poor quality of cancer assessment and recording, particularly when they are recognized as environmentally or occupationally related. This hypothesis is supported by the low agreement between histopathology, medical records, and death certificates found in Brazil [87] and Mexico [45]. In many LA countries, work-relatedness of diseases such as cancer is not commonly identified and registered in clinical settings, which reduces the number of notified cases, making prevention even more difficult [89].

In Brazil, a long battle promoted by activists, academics, and health professionals to raise awareness regarding the health effects of asbestos and the economic feasibility of alternative fibres usage culminated in 2017 with the Supreme Court admitting the validity of state laws prohibiting the use of asbestos and declaring the federal law that permitted chrysotile use unconstitutional, thus extending the prohibition for producing, transporting, transforming, and exporting the fibre for the whole country. At present the decision awaits the judgment of the appeals filed by the asbestos industry.

## Exposure assessment and prevention principles

The measurement and evaluation of exposure to asbestos is a complex activity that demands appropriate instrumentation and training, along with extensive experience [90]. It is based on the counting of air-dispersed fibres by phase contrast microscopy (PCM) and scanning electron microscopy (SEM), or Transmission Electron Microscopy (TEM), following filtration of contaminated air onto an appropriate membrane where fibres may be deposited and counted. In the case of PCM, all fibres that are regular-sized (diameter < 3μ; length > 5μ; d:l > 3:1) may be measured; with SEM, although at greater cost, there is the added possibility of identifying fibres belonging to different types of asbestos [91].

Well-established protocols are available, such as the counting of fibers under PCM by the WHO [92], and also other microscopic methods that allow for analytic activity and the recognition and identification of materials containing asbestos [93]. It is strongly recommended that any laboratory concerned with asbestos joins a network of appropriate institutes that offers the possibility of blind testing of samples in order to verify and continually improve the quality of its results [94,95].

In LA countries, laboratory capabilities to perform quantitative fibre analysis are more than scarce. Inquiries made in Argentina, Brazil, and Colombia disclosed two laboratories offering asbestos fibre counts in Argentina, one in Brazil, and two in Colombia, all doing PCM only. One of the Colombian laboratories is accredited by a national accreditation system and only the Brazilian laboratory has international accreditation. There is no expertise for doing SEM or TEM in these countries.

Systematic and continuous assessment of environmental or occupational exposures, whether qualitative or quantitative, is rarely available in low- or middle-income areas, such as LA countries. Workplaces are commonly private, monitoring of occupational exposures is left to the employers, theoretically under state supervision, and data are not usually open to the public. Often, researchers are not allowed to access or use them in studies. Examples of quantitative fibre measurements can be found in Brazil for sealing cord and gasket production workers [96] and asbestos mining and milling [97], both showing levels well above 0.1 f/ml. In Colombia, exposure assessment and cross-sectional studies conducted in workers from brake [62,63,98,99] and transmission repair shops [100] have found high asbestos exposures. In fact, in 25% of the 103 full-shift personal asbestos concentrations in 18 brake repair shops, the occupational limit of 0.1 f/cc was exceeded.

In addition to routine monitoring in work settings, exposure data are relevant to epidemiological research. According to the study design, “exposure data” could mean environmental concentration of asbestos, amount of asbestos used, attribution based on job-exposure matrices (JEM), number of exposed workers, or nominal roaster of workers. In studies addressing the work environment, it is common that exposure has to be assessed retrospectively.

Among the few occupational epidemiological studies carried out in LA, Algranti et al. [101], describing non-malignant ARDs in former A/C workers, employed a semi-quantitative index of exposure taking into account job descriptions, relative exposure weights, and number of years in each job to estimate individual exposures. In a Mexican mesothelioma case-control study, Aguilar-Madrid et al. [45], used hygienists’ expert assessments to retrospectively estimate exposure. These studies were limited to selected sectors of the population and unable to provide a gross estimate of asbestos exposure in LA countries.

In industrialized countries, several JEMs have been developed to help in the estimate of the number or prevalence of exposed workers, levels of intensity, duration, and cumulative doses, among other useful data. In JEMs, available exposure data for workers of selected industries of specific trades and occupations within a given period of time are used to project exposure estimates. This exercise, obviously, requires some assumptions. Pasetto et al. [19], used the Cancer Exposure (CAREX) database to calculate the proportion of exposed workers to asbestos by trade in Argentina, Brazil, Colombia, and Mexico. In LA countries, the scarcity of data led researchers to use asbestos consumption calculated from the volume of asbestos extraction, trade, imports, and exports as aggregated proxy information on exposure to be used in descriptive [72] or ecological analysis [82].

It is important to emphasize that the principal objective of measurement is not merely to quantify the level of exposure but rather to verify that adequate procedures are in place to protect people from asbestos and prevent the risk of inhalation of fibres. Consequently, measures serve not just to verify conformity with established norms but, above all, to make decisions about necessary precautions and safety procedures in order to avoid health risks. In industrialized countries, the rationale for such decisions is well established in most industrial processes containing asbestos, as are the most appropriate measures that need to be taken in order to eliminate risks, starting with the protection of respiratory pathways and the use of protective work clothes that prevent spreading asbestos fibres outside the contaminated area. Thus, public authorities in industrializing countries have ample opportunities for identifying tools for ARD prevention.

## Asbestos-contaminated communities

Health risks related to asbestos exposure do not concern only workers. Their family members and residents living close to asbestos polluting sources (e.g., mines or A/C plants) are also exposed to the risk of developing ARDs, as epidemiological studies have been showing since the 1960s [102,103,104]. Moreover, transnational dynamics characterizing the “politics of asbestos” [105] influenced the local management of A/C plants around the world and favored habits that can be related to the expansion of asbestos contamination outside the working place. For instance, in various geographical and socio-political contexts, it has been documented that workers and citizens could obtain, for domestic and/or public uses, asbestos residual material for free or low costs [106]. Workers were also allowed to come back home still wearing dusty work clothes, which, in the majority of cases, were washed by their wives, who themselves became exposed and contaminated [107].

In Italy, where asbestos has been prohibited since 1992, the National Register of Mesotheliomas (ReNaM) reports that in 1998–2003, out of 12,065 investigated cases, 530 individuals died from malignant mesothelioma contracted because of familial exposure, 514 from environmental exposure, and 188 from exposure during hobby-related activities [103]. In particular, contexts characterized by the presence of large A/C plants that had been operative for decades have the highest incidence of mesothelioma among citizens who never had occupational contact with asbestos. An ethnographic study conducted in Bari (Southern Italy), where the A/C plant Fibronit was operative from 1935 to 1989, has shown that the absence of risk perception of asbestos dangers among the general citizenry and in public discourses worsened the seriousness of contamination and prolonged its impact on citizens’ health [108].

Communities can also be affected because of the legacy of asbestos use, since ACPs can be present at their residences or workplaces. The peak of asbestos use in the world was reached in 1977 at 4.7 million tons/year, a volume that was gradually reduced to 2 million tons/year in the late 1990s and has remained at this level since then [9,109,110]. This means that at a global scale over the last 15 years, approximately 30 million tons of asbestos was used. This issue is especially important for non-friable ACPs, which were and in some countries still are used in the construction of different facilities such as residences, schools, offices, and hospitals. Since the asbestos encapsulation matrix can degrade releasing the fibres [61], countries are facing a complex problem that involves continuously monitoring the condition of ACPs and replacing and properly disposing deteriorated ACPs when required. Thus, an overwhelming technical challenge has been created, which will be extremely costly to implement and solve.

It is also important to explain that asbestos health risks in occupational settings are not limited to workers employed by either asbestos mines [26,111,112,113] or ACP manufacturing facilities [27,29,101,114]. A large body of scientific evidence shows that workers in other occupations are also at risk when they have to interact with ACPs that have been previously installed, and in some cases the use of these ACPs are not directly related to the occupation of these workers (i.e., secondary asbestos exposures). Workers that conduct renovations and maintenance of buildings, including electricians [115,116,117], welders [118], plumbers [119], insulators [120], sheet metal workers [120,121], building maintenance personnel [122], and seafarers [123], are examples of occupations in which these secondary exposures may occur. In other occupations, such as construction workers and auto mechanics, exposures occur because workers directly use ACPs [98,100,116,122,123,124,125,126,127]. It is important to highlight that such exposures can occur as the result of the manipulation of non-friable ACPs, something that has been extensively documented for auto mechanics, demonstrating that the encapsulation of the asbestos fibre in a matrix does not guarantee that exposure will not happen [62,98].

The impact of non-occupational (e.g., domestic or environmental) exposure to asbestos on public health needs to be further investigated to increase the reliability of epidemiological evidence of ARDs affecting social actors that did not have occupational asbestos exposure or whose history of exposure is difficult to retrace. Factors such as the long latency period of mesothelioma, the multiple causes related to a cancer’s onset, and the lack of adequate training for health professionals to recognize ARDs can favor the underdiagnosis phenomenon, and this especially occurs in contexts where the asbestos market has a strong impact on the local social fabric [7,105]. The consequent invisibility of ARDs and deaths in official health statistics worsens the effects of asbestos contamination on exposed communities by not recognizing their suffering and their rights to live in a safe environment and to have access to adequate health care practices. To break such invisibility, sufferers from ARDs have mobilized and are still mobilizing in various contexts. In this regard, a multi-sited ethnography conducted in Italy and Brazil, in two urban contexts seriously affected by the impact of asbestos manufacturing at the largest A/C plants of the Eternit label in Europe and LA, has investigated the active role of the victims, organized in associations. The victims’ actions have been fundamental to increase biomedical knowledge and to improve epidemiological evidence about the impact of asbestos exposure on health. By using their bodies as “tools to politically act” in the world [127], victims entered the processes of policy-making and knowledge production. Scientific truths are not absolute [128] and systems like that of biomedical knowledge are immersed in the social, economic, and cultural contexts that produce them [129], as the current debate in the international biomedical community about ARDs reminds us [130,131]. Accordingly, it is important to reflect on the importance of carrying on and promoting the dialogue between social actors directly affected by the impact of asbestos exposure on their lives, and the exponents of scientific knowledge appointed to investigate such an impact. This reflection inevitably concerns the scientific and ethical responsibility of professionals involved in global public health issues.

In summary, asbestos use can affect communities and workers not directly involved in the asbestos industry, creating a global public health problem. Efforts should be made to properly address the risks and adverse health consequences experienced by members of the general population, in both countries that use and countries that have banned asbestos.

## Concluding remarks

We have focused on persisting interests supporting the huge consumption of asbestos in LA, considering the large number of enterprises from industrial and trade sectors involved in asbestos-related activities in the last decades.

Worldwide, the asbestos industry has encouraged “product defense science” intended to minimize asbestos risks [130]. As a consequence, the WHO campaign to stop all use of asbestos is undermined [132]. In LA, as elsewhere, the huge economic interests underlying the extraction, trade and use of asbestos can bring about conflicts of interests within the local public health and scientific milieus. Indeed, the circumstances in which the latter have come to the surface are limited, because of the trivial number of published LA studies on asbestos-related health risks [133]. A recent review of papers published between 2010 and 2015 on asbestos-associated health risks [59] has analyzed authors’ attitudes with regard to the ritual statement on their conflicts of interest: out of six studies from LA countries, three did not include any such statement, and two and one reported lack and presence of conflicts of interest, respectively.

Conversely, concern is created by the apparent asbestos innocuous notion stemming from studies on the health impact of asbestos in LA carried out by scientists associated with the asbestos industry. Ilgren and coauthors, for instance, claimed that Bolivian crocidolite is non-carcinogenic on the basis of inappropriate procedures and flawed arguments, stating as a possible proof of harmlessness the low incidence of mesothelioma, without comments on competitive causes of death, low population life expectancy, and limited medical resources for disease recognition in the region [134]. Authors’ competing interests were not mentioned in the original paper and were revealed only in a subsequent “erratum” [135], upon pressing requests from a number of scientists [136].

Bernstein and coworkers have also repeatedly minimized chrysotile risks in the scientific literature [137,138]. They showed that inhaled Brazilian chrysotile is cleared from the rat’s lung faster than amphiboles [137]. Further, in subchronic experiments in rats, inhaled chrysotile produced less severe changes than amphiboles [138]. This has led the authors to maintain that long-lasting inhalation of chrysotile does not create a cancer risk in humans, an interpretation at odds with the epidemiological evidence as well as the results of long-term experiments in animals upon which the International Agency for Research on Cancer has produced its evaluations since 1977 [3]. These studies [137,138] were supported by SAMA, the enterprise owning the Brazilian chrysotile mines, and lacked any allusion to the authors’ conflicts of interest. In 2013, Bernstein and others coauthored a review on chrysotile risks, supported by a grant from the International Chrysotile Association in cooperation with the Canadian Chrysotile Association, which concluded that low exposures to chrysotile do not present a detectable risk to health [24].

In LA countries, scientific papers such as those mentioned above may convey unwarranted consideration to the public opinion through the convergence between scientists and journalists. For instance, in 2012, *El Espectador*, one of the major Colombian newspapers, reported that the most rigorous studies show that chrysotile is not carcinogenic [139]. In order to prevent distorting messages addressed to the public, independent studies need to be developed, and there must be competent and well-informed researchers and public health professionals with a strong reference to professional ethics and personal integrity.

In this perspective, we have highlighted the importance and urgency for adopting prevention measures supported by effective policies taking into account the following arguments:

- In countries in which asbestos use is not yet either forbidden or subject to control, a major aim is the creation of a high degree of understanding and awareness that may lead to the adoption of a total ban. However, it is worth emphasizing that, while a prohibition on the use of asbestos is necessary, it is not sufficient [140]. For decades we will continue to live with buildings and workplaces that contain this material, often in a state of degradation and with a consequent release of fibres into the air. This risk demands, above all, the mapping and, when necessary, proper decontamination of such places.
- The apparent low occurrence of mesothelioma cases in LA countries should be seen in the context of probable underreporting due to diagnostic limitations, weaknesses in epidemiologic surveillance, and competitive causes of death. The absence of reliable data should not be used as a reason for delaying regulatory interventions.
- Local economy alarming for disadvantages, motivated by closing asbestos-related activities, should not ignore the economic and social/human costs resulting from ARDs for the entire society, and thus claim multi-sectoral regulatory interventions.
- Dialogue between the asbestos-exposed communities, including individuals experiencing the health impact and researchers, has to be strengthened and fostered according to both local needs/priorities and ethical responsibility. In this frame, the convergence of forces from researchers and public health professionals, associations of formerly exposed workers, and asbestos victims has been motivating and supporting many judicial proceedings.

As an international multidisciplinary network of professionals involved in the global public health issues of asbestos, we have discussed in the present paper critical issues from the scientific, socio-economic, and epidemiological points of view, which are currently debated in many LA countries still using asbestos. In recent years, this international scientific cooperation network has been supporting skills and energies at national level for asbestos risk prevention in LA. The associated aim is to contribute to contrast health inequities between countries related to the unjust and avoidable burden of ARD and the related socio-economic impacts, within a global public health perspective.

## References

[1] Word Health Organization. Asbestos: Elimination of asbestos-related diseases. Fact sheet. Updated 6 2016 Available at: http://www.who.int/mediacentre/factsheets/fs343/en/. Accessed October 31, 2017.

[2] World Health Organization. Chrysotile Asbestos Geneva: WHO; 2014 Available at: http://www.who.int/ipcs/assessment/public_health/chrysotile_asbestos_summary.pdf. Accessed August 25, 2017.

[3] International Agency for Research on Cancer. IARC Monographs: Arsenic, metals, fibres and dusts. Volume 100C. A review of human carcinogens; 2012 Available at: http://monographs.iarc.fr/ENG/Monographs/vol100C/mono100C.pdf. Accessed May 10, 2017.

[4] World Health Organization. Outline for the development of national programmes for elimination of asbestos-related diseases WHO-ILO; 2007 Available at: http://www.who.int/occupational_health/publications/asbestosdoc/en/. Accessed October 14, 2017.

[5] Ogunseitan OA. The asbestos paradox: Global gaps in the translational science of disease prevention. Bull Word Health Organ. 2015; 93(5): 359–360. DOI: 10.2471/BLT.14.142307 PMC4510811 26229210

[6] Collegium Ramazzini. The Global Health Dimensions of Asbestos and Asbestos-related Diseases; 2015 6 24 Available at: http://www.collegiumramazzini.org/news1.asp?id=130. Accessed October 15, 2017. 10.1539/joh.16-2002-ST PMC5356970 27040479

[7] Marsili D, Terracini B, Santana VS, et al. Prevention of asbestos-related disease in countries currently using asbestos. Int J Envir Res Health. 2016; 13(5): 494 DOI: 10.3390/ijerph13050494 PMC4881119 27187433

[8] Marsili D, Comba P, Pasetto R and Terracini B. International scientific cooperation on asbestos related disease prevention in Latin America. Ann Global Health. 2014; 80(4): 247–50. DOI: 10.1016/j.aogh.2014.09.002 25459324

[9] Marsili D and Comba P. Asbestos case and its current implications for global health. Ann Ist Super Sanità. 2013; 49(3): 249–51. 24071603 10.4415/ANN_13_03_03

[10] Frank AL and Joshi TK. The global spread of asbestos. Ann Global Health. 2014; 80: 257–62. DOI: 10.1016/j.aogh.2014.09.016 25459326

[11] Egilman D, Bird T and Lee C. Dust diseases and the legacy of corporate manipulation of science and Law. Int J Occup Environ Health. 2014; 20(2): 115–25. DOI: 10.1179/1077352514Z.000000000104 24999846 PMC4090870

[12] Castleman B. The export of hazardous industries in 2015. Environmental Health. 2016; 15(8): 1–6. DOI: 10.1186/s12940-016-0091-6 26786721 PMC4717658

[13] U.S. Geological Survey. Minerals yearbook 2005: Asbestos. Available at: http://minerals.usgs.gov/minerals/pubs/commodity/asbestos/asbesmyb05.pdf. Accessed July 15, 2017.

[14] U.S. Geological Survey. Minerals yearbook 2014: Asbestos. Available at: http://minerals.usgs.gov/minerals/pubs/commodity/asbestos/myb1-2014-asbes.pdf. Accessed July 15, 2017.

[15] U.S. Geological Survey. Mineral Commodity Summaries 2016. Available at: http://minerals.usgs.gov/minerals/pubs/commodity/asbestos/mcs-2016-asbes.pdf. Accessed July 15, 2017.

[16] Markowitz S. Cancer of the respiratory tract due to asbestos and zeolites In: Parkes’ Occupational lung disorders. 2017; 259–76. Taylor and Francis.

[17] Van der Bij S, Vermeulen RC, Portengen L, Moons KG and Koffijberg H. Expected number of asbestos-related lung cancers in the Netherlands in the next two decades: A comparison of methods. Occup Environ Med. 2016; 73(5): 342–9. DOI: 10.1136/oemed-2014-102614 26858099

[18] McCormack V, Peto J, Byrnes G, Straif K and Boffetta P. Estimating the asbestos-related lung cancer burden from mesothelioma mortality. Br J Cancer. 2012; 106(3): 575–84. DOI: 10.1038/bjc.2011.563 22233924 PMC3273352

[19] Pasetto R, Terracini B, Marsili D and Comba P. Occupational burden of asbestos-related cancer in Argentina, Brazil, Colombia, and Mexico. Ann Glob Health. 2014; 80(4): 263–8. DOI: 10.1016/j.aogh.2014.09.003 25459327

[20] Rushton L, Hutchings S and Brown T. The burden of cancer at work: Estimation as the first step to prevention. Occup Environ Med. 2008; 65(12): 789–800. DOI: 10.1136/oem.2007.037002 18079154

[21] Lenters V, Vermeulen R, Dogger S, et al. A meta-analysis of asbestos and lung cancer: Is better quality exposure assessment associated with steeper slopes of the exposure–response relationships? Environ Health Perspect. 2011; 119(11): 1547–55. DOI: 10.1289/ehp.1002879 21708512 PMC3226488

[22] Pira E, Romano C, Donato F, Pelucchi C, Vecchia C and Boffetta P. Mortality from cancer and other causes among Italian chrysotile asbestos miners. Occup Environ Med. 2017; 74(8): 558–563. DOI: 10.1136/oemed-2016-103673 28438787

[23] Yarborough CM. The risk of mesothelioma from exposure to chrysotile asbestos. Curr Opin Pulm Med. 2007; 13(4): 334–8. DOI: 10.1097/MCP.0b013e328121446c 17534182

[24] Bernstein D, Dunnigan J, Hesterberg T, et al. Health risk of chrysotile revisited. Crit Rev Toxicol. 2013; 43: 154–183. DOI: 10.3109/10408444.2012.756454 23346982 PMC3581056

[25] Feder I, Tischoff I, Theile A, et al. The asbestos fibre burden in human lungs: New insights into the chrysotile debate. Euro Respir J. 2017; 49(6): 1160–2534. DOI: 10.1183/13993003.02534-2016 PMC5898940 28663314

[26] Liddell FD, McDonald AD and McDonald JC. The 1891–1920 birth cohort of Quebec chrysotile miners and millers: Development from 1904 and mortality to 1992. Ann Occup Hyg. 1997; 41(1): 13–36. DOI: 10.1016/S0003-4878(96)00044-0 9072947

[27] Hein MJ, Stayner LT, Lehman E and Dement JM. Follow-up study of chrysotile textile workers: Cohort mortality and exposure-response. Occup Environ Med. 2007; 64(9): 616–25. DOI: 10.1136/oem.2006.031005 17449563 PMC2092560

[28] Elliott L, Loomis D, Dement J, Hein MJ, Richardson D and Stayner L. Lung cancer mortality in North Carolina and South Carolina chrysotile asbestos textile workers. Occup Environ Med. 2012; 69: 385–90. DOI: 10.1136/oemed-2011-100229 22267448

[29] Wang X, Lin S, Yano E, et al. Exposure-specific lung cancer risks in Chinese chrysotile textile workers and mining workers. Lung Cancer. 2014; 85: 119–24. DOI: 10.1016/j.lungcan.2014.04.011 24854404

[30] Deng, Q, Wang X, Wang M and Lan Y. Exposure-response relationship between exposure and mortality from lung cancer and asbestosis. Occup Environ Med. 2012; 69(2): 81–6. DOI: 10.1136/oem.2011.064899 21742741

[31] Courtice MN, Wang X, Lin S, Yu IT, Berman DW and Yano E. Exposure-response estimate for lung cancer and asbestosis in a predominantly chrysotile-exposed Chinese factory cohort. Am J Ind Med. 2016; 59(5): 369–78. DOI: 10.1002/ajim.22579 26969815

[32] van der Bij S, Koffijberg H, Lenters V, et al. Lung cancer risk at low cumulative asbestos exposure: Meta-regression of the exposure-response relationship. Cancer Causes Control. 2013; 24(1): 1–12. DOI: 10.1007/s10552-012-0107-7 23187858

[33] Olsson AC, Vermeulen R, Schüz J, et al. Exposure-response analyses of asbestos and lung cancer subtypes in a pooled analysis of case-control studies. Epidemiology. 2017; 28(2): 288–99. DOI: 10.1097/EDE.0000000000000604 28141674 PMC5287435

[34] Markowitz SB, Levin SM, Miller A and Morabia A. Asbestos, asbestosis, smoking, and lung cancer. New findings from the North American insulator cohort. Am J Respir Crit Care Med. 2013; 188(1): 90–6. DOI: 10.1164/rccm.201302-0257OC 23590275

[35] Nielsen LS, Bælum J, Rasmussen J, et al. Occupational asbestos exposure and lung cancer—A systematic review of the literature. Arch Environ Occup Health. 2014; 69(4): 191–206. DOI: 10.1080/19338244.2013.863752 24410115

[36] Offermans NS, Vermeulen R, Burdorf A, et al. Occupational asbestos exposure and the risk of esophageal, gastric and colorectal cancer in the prospective Netherlands Cohort Study. Int J Cancer. 2014; 135(8): 1970–77. DOI: 10.1002/ijc.28817 24585528

[37] Lin S, Wang X, Yano E, et al. Exposure to chrysotile mining dust and digestive cancer mortality in a Chinese miner/miller cohort. Occup Environ Med. 2014; 71(5): 323–8. DOI: 10.1136/oemed-2013-101360 24436059

[38] Clin B, Morlais F, Launoy G, et al. Cancer incidence within a cohort occupational exposed to asbestos: A study of dose-response relationships. Occup Environ Med. 2011; 68(11): 832–6. DOI: 10.1136/oem.2010.059790 21406385

[39] Boulanger M, Morlais F, Bouvier V, et al. Digestive cancers and occupational asbestos exposure: Incidence study in a cohort of asbestos plant workers. Occup Environ Med. 2015; 72(11): 792–7. DOI: 10.1136/oemed-2015-102871 26304776

[40] Paris C, Thaon I, Hérin F, et al. Occupational asbestos exposure and incidence of colon and rectal cancers in French men: The asbestos-related diseases cohort (ARDCo-Nut). Environ Health Perspect. 2017; 125(3): 409–15. DOI: 10.1289/EHP153 27517294 PMC5332175

[41] Ferrante D, Chellini E, Merler E, et al. Italian pool of asbestos workers cohorts: Mortality trends of asbestos-related neoplasms after long time since first exposure. Occup Environ Med. 2017; 74: 887–898. DOI: 10.1136/oemed-2016-104100 28775133

[42] Virta RL. Worldwide asbestos supply and consumption trends from 1900 through 2003 Circular 1298. Denver: U.S. Department of the Interior, U.S. Geological Survey; 2006.

[43] Harington JS, McGlashan ND and Chelkowska EZ. South Africa’s export trade in asbestos: Demise of an industry. Am J Ind Med. 2010; 53: 524–34. 19943319 10.1002/ajim.20784

[44] Aguilar-Madrid G, Juárez-Pérez CA, Markowitz S, Hernández-Avila M, Sanchez Roman FR and Vázquez Grameix JH. Globalization and the transfer of hazardous industry: Asbestos in Mexico, 1979–2000. Int J Occup Environ Health. 2003; 9(3): 272–9. DOI: 10.1179/oeh.2003.9.3.272 12967165

[45] Aguilar-Madrid G, Robles-Pérez E, Juárez-Pérez CA, Alvarado-Cabrero I, Rico-Méndez FG and Javier KG. Case-control study of pleural mesothelioma in workers with social security in Mexico. Am J Ind Med. 2010; 53(3): 241–51. 20017186 10.1002/ajim.20780

[46] Kazan-Allen L. Asbestos en Colombia. Available at: http://www.ibasecretariat.org/lka-asbestos-in-colombia-2012.php. Accessed March 16, 2017.

[47] Hernandez Dias M. Plan nacional para la prevencion de la silicosis, la neumoconiosis de los mineros de carbon y la asbestosis 2010–2030 MPS 326 de 2009. Bogotá: Ministerio de la Proteccion Social; 2010.

[48] Concepto jurídico al proyecto de Ley 97 de 2015 Senado. Concepto de Ascolfibras del proyecto de Ley Número 97 de 2015 Senado. Available at: http://www.imprenta.gov.co/gacetap/gaceta.mostrar_documento?p_tipo=2038&p_numero=97&p_consec=44537#_ftn19. Accessed March 16, 2017.

[49] Brazil. Ministry of Labour. Registration: Benzene and asbestos. Available at: http://trabalho.gov.br/seguranca-e-saude-no-trabalho/cadastro-benzeno-e-asbesto. Accessed May 12, 2017.

[50] Federação das Indústrias do Estado de São Paulo – FIESP. O papel dos produtos de amianto na cadeia da construção civil. Dimensão econômica e efeitos concorrenciais São Paulo: Departamento da Indústria da Construção – DECONCIC, FIESP; 2009.

[51] Gonçalves da Silva AL and Etulain CA. The economic impact of the banning of the use of asbestos in Brazil. Available at: http://ibasecretariat.org/alg-cre-econ-impact-ban-asbestos-brazil.pdf. Accessed December 16, 2016.

[52] Allen LP, Jorge Baez J, Stern MEC and George F. Asbestos economic assessment of bans and declining production and consumption. Available at: http://www.euro.who.int/__data/assets/pdf_file/0009/341757/Asbestos_EN_WEB_reduced.pdf?ua=1. Accessed June 24, 2017.

[53] World Health Organization, Regional Office for Europe. The Human and Financial Burden of Asbestos in the WHO European Region. Meeting Report 5–6 November 2012, Bonn, Germany; 2013 Available at: http://www.euro.who.int/__data/assets/pdf_file/0003/194133/RB-Asbestos-Mtg-Report-Bonn-2012.pdf?ua=1. Accessed March 24, 2017.

[54] Chrysotile Institute. Available at: http://hwww.chrysotile.com/en/about.aspx. Accessed March 24, 2017.

[55] International Chrysotile Association. Available at: http://www.chrysotileassociation.com/en/about.php. Accessed March 24, 2017.

[56] Altman DG and Bland JM. Absence of evidence is not evidence of absence. BMJ. 1995; 311(7003): 485 DOI: 10.1136/bmj.311.7003.485 7647644 PMC2550545

[57] Alderson P. Absence of evidence is not evidence of absence. BMJ. 2004; 328(7438): 476–7. DOI: 10.1136/bmj.328.7438.476 14988165 PMC351831

[58] Fino C. El Senado de las Causas Sociales y la Reconciliación. “Sobre el asbestos, hay que legislar con rigor: Senadora Sofía Gaviria”. Available at: http://www.senado.gov.co/historia/item/24764-sobre-el-asbesto-hay-que-legislar-con-rigor-senadora-sofia-gaviria. Accessed March 16, 2017.

[59] Valenzuela M, Giraldo M, Gallo-Murcia S, Pineda J, Santos L and Ramos-Bonilla JP. Recent scientific evidence regarding asbestos use and health consequences of asbestos exposure. Curr Environ Health Rep. 2016; 3(4): 335–47. DOI: 10.1007/s40572-016-0109-9 27696225

[60] International Ban Asbestos Secretariat. Available at: http://www.ibasecretariat.org/alpha_ban_list.php. Accessed March 17, 2017.

[61] Campopiano A, Ramires D, Zakrzewska AM, Ferri R, D’annibale A and Pizzutelli G. Risk assessment of the decay of asbestos cement roofs. Ann Occup Hyg. 2009; 53(6): 627–38. DOI: 10.1093/annhyg/mep036 19491148

[62] Cely-García MF, Curriero FC, Giraldo M, et al. Factors associated with non-compliance of asbestos occupational standards in brake repair workers. Ann Occup Hyg. 2016; 60(8): 1020–35. DOI: 10.1093/annhyg/mew028 27234376

[63] Cely-García MF, Sánchez M, Breysee P and Ramos-Bonilla JP. Personal exposure to asbestos fibers during brake maintenance of passenger vehicles. Ann Occup Hyg. 2012; 56(9): 985–99. 22926785 10.1093/annhyg/mes030

[64] Terracini B, Mirabelli D, Baur X, Landrigan P and Collegium Ramazzini. Comments on the causation of malignant mesothelioma: Rebutting the false concept that recent exposures to asbestos do not contribute to causation of mesothelioma. Am J Ind Med. 2016; 59(6): 506–7. DOI: 10.1002/ajim.22590 27094688

[65] Asbestos, asbestosis, and cancer: The Helsinki criteria for diagnosis and attribution. Scand J Work Environ Health. 1997; 23(4): 311–6. 9322824

[66] Wolff H, Vehmas T, Oksa P, Rantanen J and Vainio H. Asbestos, asbestosis, and cancer, the Helsinki criteria for diagnosis and attribution 2014: Recommendations. Scand J Work Environ Health. 2015; 41(1): 5–15. DOI: 10.5271/sjweh.3462 25299403

[67] Merler E, Somigliana A, Girardi P and Barbieri PG. Residual fibre lung burden among patients with pleural mesothelioma who have been occupationally exposed to asbestos. Occup Environ Med. 2017; 74(3): 218–27. DOI: 10.1136/oemed-2015-103382 27821674

[68] Magnani C, Fubini B, Mirabelli D, et al. Pleural mesothelioma: Epidemiological and public health issues. Report from the second Italian consensus conference on pleural mesothelioma. Med Lav. 2013; 104(3): 191–202. 23879063

[69] Ugolini D, Bonassi S, Cristaudo A, Leoncini G, Battista Ratto G and Neri M. Temporal trend, geographic distribution, and publication quality in asbestos research. Environ Sci Pollut Res Int. 2014; 22(9): 6957–67. DOI: 10.1007/s11356-014-3925-1 25475619

[70] Méndez Vargas MM, Maldonado Torres L, Stanislawski EC and Mendoza Ugalde HA. Mesotelioma maligno en un trabajador del asbesto. Revista Médica. 1982; 20(3): 249–57.

[71] De Capitani EM, Metze K, Frazzato C, et al. Mesotelioma maligno de pleura com associação etiológica a asbesto: A propósito de três casos clínicos. Rev Ass Med Brasil. 1997; 43(3): 265–72. DOI: 10.1590/S0104-42301997000300015 9497555

[72] Rodríguez EJ. Asbestos banned in Argentina. Int J Occup Environ Health. 2004; 10(2): 202–8. DOI: 10.1179/oeh.2004.10.2.202 15281380

[73] Castro HC, Ribeiro TE and Gonçalves KS. Doença relacionada ao asbesto: Estudo de sete casos em duas famílias. Pulmão RJ. 2007; 16(1): 44–8.

[74] Maciel JGFS, Carneiro APS, Fiorentini L, et al. Mesotelioma maligno de pleura e exposição ocupacional ao asbesto: Relato de dois casos diagnosticados em Belo Horizonte, Minas Gerais. Rev Med Minas Gerais. 2010; 20(Suppl 2): S94–S98.

[75] Barrera RR, Chavarria JG and Morales JF. Mesotelioma maligno: Experiência clinico-patológica de 247 casos. Rev Chil Enf Respir. 2010; 26: 134–40. DOI: 10.4067/S0717-73482010000300003

[76] Matos GR and Pinheiro RDC. Perfil dos óbitos por mesotelioma registrados no sistema de informação de mortalidade em Santa Catarina, no período de 1998–2009. Rev Saúde Públ Santa Cat Florianópolis. 2011; 4(1).

[77] Zurbiggren R and Capone L. Enfermedad pulmonar por amianto en trabajadores de acería. Medicina (Buenos Aires). 2013; 73: 224–30. 23732197

[78] Standen CSJ and Cuevas TM. Fibras grises de muerte: El silencio del mayor genocidio industrial de Chile Santiago: Global Greengrants Funds & Unidos contra El asbesto; 2013.

[79] Nishikawa K, Takahashi K, Karjalainen A, et al. Recent mortality from pleural mesothelioma, historical patterns of asbestos use, and adoption of bans: A global assessment. Environ Health Perspect. 2008; 116(12): 1675–80. DOI: 10.1289/ehp.11272 19079719 PMC2599762

[80] Pedra F, Tambellini AT, de Pereira BB, da Costa AC and de Castro HA. Mesothelioma mortality in Brazil, 1980–2003. Int J Occup Environ Health. 2008; 14: 70–75. DOI: 10.1179/oeh.2008.14.3.170 18686716

[81] Pedra F, Silva P, Matos I and Castro H. Mesothelioma mortality rate in Brazil, 1980 to 2010. Rev Bras Cancerol. 2014; 60(3): 199–206.

[82] Algranti E, Saito CA, Carneiro AP, Moreira B, Mendonca EM and Bussacos MA. The next mesothelioma wave: Mortality trends and forecast to 2030 in Brazil. Cancer Epidemiol. 2015; 39(5): 687–92. DOI: 10.1016/j.canep.2015.08.007 26320384

[83] Park EK, Takahashi K, Hoshuyama T, et al. Global magnitude of reported and unreported mesothelioma. Environ Health Perspect. 2011; 119(4): 514–8 DOI: 10.1289/ehp.1002845 21463977 PMC3080934

[84] Trotta A, Santana VS and Alazraqui M. Mortalidad por mesotelioma en Argentina, 1980–2013. Salud Colectiva. 2017; 13(1): 35–44. DOI: 10.18294/sc.2017.1027 28562724

[85] Trotta A, Santana VS and Andreozzi L. Forecasting of mesothelioma mortality in Argentina, 2014–2023. Occup Environ Med. 2016; 73(Suppl 1): A1.

[86] Pinheiro GA, Antão VC, Monteiro MMT, Capelozzi VL and Terra-Filho M. Mortality from pleural mesothelioma in Rio de Janeiro, Brazil, 1979–2000: Estimation from death certificates, hospital records, and histopathologic assessments. Int J Occup Environ Health. 2003; 9(2): 147–52. DOI: 10.1179/oeh.2003.9.2.147 12848243

[87] Driscoll T, Steenland K, Pruss Ustun A, Nelson DI and Leigh J. Occupational carcinogens: Assessing the environmental burden of disease at national and local levels Geneva: WHO; 2004. (environmental Burden of Disease Series, No 6).

[88] Frank A. Why countries ban asbestos: Some alternative thoughts. Int J Occup Environ Health. 2013; 19(2): 136 DOI: 10.1179/1077352513Z.00000000060 23684272

[89] Santana VS and Ribeiro Netto FS. Occupational cancer burden in developing countries and the problem of informal workers. Environmental Health. 2011; 10(Suppl 1): S10. 21489206 10.1186/1476-069X-10-S1-S10 PMC3073188

[90] Cavariani F, Marconi A and Sala O. Asbestos: Sampling, analytical techniques and limit values. Ital Occup Environ Hyg. 2010; 1(1): 18–28.

[91] International Organizational for Standardization. ISO 14966:2002: Ambient air – Determination of numerical concentration of inorganic fibrous particles – Scanning electron microscopy method Geneva: ISO; 2002.

[92] World Health Organization. Determination of airborne fibre number concentrations. A recommended method by phase-contrast optical microscopy (membrane filter method) WHO: Geneva; 1977.

[93] Health and Safety Executive (HSE). Method for the determination of hazardous substances: Asbestos in bulk materials. MDHS 77; 6 1994.

[94] Crawford NP, Brown P and Cowie AJ. The Rice and Africa schemes for asbestos fibre counting. Ann Occup Hyg. 1992; 36(1): 59–69.

[95] National Voluntary Laboratory Accreditation Program (NVLAP). Asbestos fiber analysis program. Available at: https://www.nist.gov/nvlap/accreditation-programs/asbestos-fiber-analysis. Accessed July 4, 2017.

[96] Freitas JFP, Scwartsman G, Colucci S, et al. Avaliação de ambientes de trabalho em trabalhadores expostos a poeira de sílica e fibras de asbesto. Rede Especial – Revista do Projeto de Cooperação Técnica Brasil-Itália São Paulo: IMESP; 1998.

[97] Bagatin E, Neder JA, Nery LE, et al. Non-malignant consequences of decreasing asbestos exposure in the Brazil chrysotile mines and mills. Occup Environ Med. 2005; 62: 381–9. DOI: 10.1136/oem.2004.016188 15901885 PMC1741034

[98] Cely-García MF, Torres-Duque C, Durán M, et al. Personal exposure to asbestos and respiratory health of heavy vehicle brake mechanics. J Expo Sci Environ Epidemiol. 2015; 25(1): 26–36. DOI: 10.1038/jes.2014.8 24496218

[99] Cely-García MF, Curriero FC, Sánchez-Silva M, et al. Estimation of personal exposure to asbestos of brake repair workers. J Expo Sci Environ Epidemiol; 2016. 10.1038/jes.2016.76 27966665

[100] Salazar N, Cely-García MF, Breysee PN and Ramos-Bonilla JP. Asbestos exposure among transmission mechanics in automotive repair shops. Ann Occup Hyg. 2015; 59(3): 292–306. 25395207 10.1093/annhyg/meu093

[101] Algranti E, Mendonça EMC, De Capitani EM, Freitas JBP, Silva HC and Bussacos MA. Non-malignant asbestos-related diseases in Brazilian asbestos-cement workers. Am J Ind Med. 2001; 40: 240–54. DOI: 10.1002/ajim.1095 11598970

[102] Selikoff IJ, Churg J and Hammond, EC. Relation between exposure to asbestos and mesothelioma. N Engl J Med. 1965; 272(11): 560–5. DOI: 10.1056/NEJM196503182721104 14248731

[103] Marinaccio A, Binazzi A, Bonafede M, et al. Malignant mesothelioma due to non-occupational asbestos exposure from the Italian National Surveillance System (ReNaM): Epidemiology and public health issues. Occup Environ Med. 2015; 72(9): 648–55. DOI: 10.1136/oemed-2014-102297 26045315

[104] Ferrante D, Mirabelli D, Tunesi S, Terracini B and Magnani C. Pleural mesothelioma and occupational and non-occupational asbestos exposure: A case-control study with quantitative risk assessment. Occup Environ Med. 2016; 73: 147–53. DOI: 10.1136/oemed-2015-102803 26265669

[105] Waldman L. The politics of asbestos. Understanding of risk, disease, and protest London: Earthscan; 2011.

[106] Rossi G. Amianto. Processo alle fabbriche della morte Milano: Melampo; 2012.

[107] Ferrante D, Bertolotti M, Todesco A, Mirabelli D, Terracini B and Magnani C. Cancer mortality and incidence of mesothelioma in a cohort of wives of asbestos workers in Casale Monferrato, Italy. Environ Health Perspect. 2007; 115(10): 1401–5. DOI: 10.1289/ehp.10195 17938727 PMC2022648

[108] Mazzeo A. Indagine antropologica sull’esperienza di malattia provocata dall’esposizione ambientale all’amianto Il caso Fibronit di Bari, master’s degree. University of Bologna; 2010.

[109] Park EK, Takahashi K, Jiang Y, Movahed M and Kameda T. Elimination of asbestos use and asbestos related diseases: An unfinished story. Cancer Sci. 2012; 103(10): 1751–5. DOI: 10.1111/j.1349-7006.2012.02366.x 22726320 PMC7659290

[110] Choi Y, Lim S and Paek D. Trades of dangers: A study of asbestos industry transfer cases in Asia. Am J Ind Med. 2013; 56(3): 335–46. DOI: 10.1002/ajim.22144 23192535

[111] Berry G, Reid A, Aboagye-Sarfo P, et al. Malignant mesotheliomas in former miners and millers of crocidolite at Wittenoom (Western Australia) after more than 50 years follow-up. Br J Cancer. 2012 2 28; 106(5): 1016–20. 22315054 10.1038/bjc.2012.23 PMC3305966

[112] Nayebzadeh A, Case BW, Massé J and Dufresne A. Mineralogical and exposure determinants of pulmonary fibrosis among Québec chrysotile miners and millers. Int Arch Occup Environ Health. 2006; 79(3): 227–36. DOI: 10.1007/s00420-005-0046-7 16283364

[113] Sluis-Cremer GK. Asbestosis in South African asbestos miners. Environ Res. 1970; 3(4): 310–9. DOI: 10.1016/0013-9351(70)90024-1 5489822

[114] Menegozzo S, Comba P, Ferrante D, et al. Mortality study in an asbestos cement factory in Naples, Italy. Ann Inst Super Sanita. 2011; 47(3): 296–304. 10.4415/ANN_11_03_10 21952156

[115] Hodgson MJ, Parkinson DK, Sabo S, Owens GR and Feist JH. Asbestosis among electricians. J Occup Med. 1988; 30(8): 638–40. DOI: 10.1097/00043764-198808000-00007 3262731

[116] Järvholm B and Englund A. The impact of asbestos exposure in Swedish construction workers. Am J Ind Med. 2014; 57(1): 49–55. DOI: 10.1002/ajim.22264 24108505

[117] García-Closas M and Christiani DC. Asbestos-related diseases in construction carpenters. Am J Ind Med. 1995; 27(1): 115–25. DOI: 10.1002/ajim.4700270111 7900729

[118] Pairon JC, Martinon L, Iwatsubo Y, et al. Retention of asbestos bodies in the lungs of welders. Am J Ind Med. 1994; 25(6): 793–804. DOI: 10.1002/ajim.4700250604 8067357

[119] Burdett G and Bard D. Exposure of UK industrial plumbers to asbestos, Part I: Monitoring of exposure using personal passive samplers. Ann Occup Hyg. 2007; 51(2): 121–30. 17189281 10.1093/annhyg/mel078

[120] Lilis R, Miller A, Godbold J, Benkert S, Wu X and Selikoff IJ. Comparative quantitative evaluation of pleural fibrosis and its effects on pulmonary function in two large asbestos-exposed occupational groups—Insulators and sheet metal workers. Environ Res. 1992; 59(1): 49–66. DOI: 10.1016/S0013-9351(05)80225-7 1425519

[121] Drucker E, Nagin D, Michaels D, Lacher M and Zoloth S. Exposure of sheet-metal workers to asbestos during the construction and renovation of commercial buildings in New York City. A case study in Social Medicine. Ann N Y Acad Sci. 1987; 502: 230–44. DOI: 10.1111/j.1749-6632.1987.tb37655.x 3477975

[122] Mlynarek S, Corn M and Blacket C. Asbestos exposure of building maintenance personnel. Regul Toxicol Pharmacol. 1996; 23(3): 213–24. DOI: 10.1006/rtph.1996.0045 8812963

[123] Saarni H, Pentti J and Pukkala E. Cancer at sea: A case-control study among male Finnish seafarers. Occup Environ Med. 2002; 59(9): 613–9. DOI: 10.1136/oem.59.9.613 12205234 PMC1740353

[124] Kishimoto T, Morinaga K and Kira S. The prevalence of pleural plaques and/or pulmonary changes among construction workers in Okayama, Japan. Am J Ind Med. 2000; 37(3): 291–5. DOI: 10.1002/(SICI)1097-0274(200003)37:3<291::AID-AJIM7>3.0.CO;2-A 10642419

[125] Kakooei H, Hormozy M and Marioryad H. Evaluation of asbestos exposure during brake repair and replacement. Industrial Health. 2011; 49(3): 374–80. DOI: 10.2486/indhealth.MS1166 21372435

[126] Ameille J, Rosenberg N, Matrat M, et al. Asbestos-related diseases in automobile mechanics. Ann Occup Hyg. 2012; 56(1): 55–60. 21965465 10.1093/annhyg/mer066 PMC3678990

[127] Mauss M. Techniques of the body. Economy and Society. 1973; 2(1): 70–88. DOI: 10.1080/03085147300000003

[128] Kuhn TS. The Structures of Scientific Revolutions Chicago: The University of Chicago Press; 1963.

[129] Kleinman A. Patients and Healers in the Context of Culture: An Exploration of the Borderland between Anthropology, Medicine, and Psychiatry Berkeley: University of California Press; 1980.

[130] Terracini B and Mirabelli D. Asbestos and product defense science. Int J Epidemiol. 2016; 45: 614–8. DOI: 10.1093/ije/dyw136 27255439

[131] Castleman B. Controlled use of asbestos. Int J Occup Environ Health. 2003; 9(3): 294–8. DOI: 10.1179/oeh.2003.9.3.294 12967168

[132] Baur X, Soskolne CL, Lemen RA, Schneider J, Woitowitz HJ and Budnik LT. How conflicted authors undermine the World Health Organization (WHO) campaign to stop all use of asbestos: Spotlight on studies showing that chrysotile is carcinogenic and facilitates other non-cancer asbestos-related diseases. Int J Occup Environ Health. 2015; 21(2): 176–9. DOI: 10.1179/2049396714Y.0000000105 25729927 PMC4457129

[133] Raj P, Hohenadel K, Demers P, Hoar Zahm S and Blair A. Published occupational cancer epidemiology research: Results from a comprehensive review of the literature. Am J Industr Med. 2014; 57: 259–64. DOI: 10.1002/ajim.22280 24488816

[134] Ilgren EB, Van Orden DR, Lee RJ, Kamiya YM and Hoskins JA. Further studies of Bolivian crocidolite—Part IV: Fibres width, fibre drift and their relation to mesothelioma induction: Preliminary findings. Epidemiol Biostat Public Health. 2015; 12(2): e11161-1–e11161-11.

[135] Erratum: Epidemiol Biostat Public Health. 2016.

[136] Magnani C, Barone-Adesi F, Biggeri A, et al. Letter to editors. Epidemiol Biostat Public Health. 2016; 13: e11590–1.

[137] Bernstein DM, Rogers R and Smith P. The biopersistence of Brazilian chrysotile asbestos following inhalation. Inhal Toxicol. 2004; 16(11–12): 745–61. DOI: 10.1080/08958370490490176 16036745

[138] Bernstein DM, Rogers R, Smith P and Chevalier J. The toxicological response of Brazilian chrysotile asbestos: A multi dose subchronic 90-day inhalation toxicology study with 92-day recovery to assess cellular and pathological response. Inhal Toxicol. 2006; 18(5): 313–32. DOI: 10.1080/08958370500515871 16513591

[139] El Espectador. 2 10, 2012 Avanza venta de Minera Las Brisas, en liquidación. Economía. Available at: http://www.elespectador.com/noticias/economia/avanza-venta-de-minera-brisas-liquidacion-articulo-327651. Accessed June 9, 2017.

[140] Marsili D, Angelini A, Bruno C, et al. Asbestos ban in Italy: A major milestone, not the final cut. Int J Environ Res Public Health. 2017; 14(11): 1379 DOI: 10.3390/ijerph14111379 PMC5708018 29137208

